# Inhibitory Activity of *Illicium verum* Extracts against Avian Viruses

**DOI:** 10.1155/2020/4594635

**Published:** 2020-01-25

**Authors:** Mohammed S. Alhajj, Mahmood A. Qasem, Saud I. Al-Mufarrej

**Affiliations:** ^1^Department of Veterinary Medicine, College of Agriculture and Veterinary Medicine, Thamar University, Dhamar, Yemen; ^2^Department of Animal Production, College of Food and Agriculture Sciences, King Saud University, Riyadh, Saudi Arabia

## Abstract

This study aimed at screening the inhibitory activity of *Illicium verum* extracts against avian reovirus, infectious bursal disease virus (IBDV), Newcastle disease virus (NDV), and infectious laryngotracheitis virus (ILTV). The cytotoxic and antiviral actions of 3 extracts, absolute methanol (100MOH), 50% methanol (50MOH), and aqueous extracts (WA.), were evaluated by MTT assay. The *Illicium verum* extracts were added to the cultured chick embryo fibroblast (CEF) with tested viruses in three attacks, preinoculation, postinoculation, and simultaneous inoculation. The three extracts showed antiviral inhibitory activity against all tested viruses during simultaneous inoculation and preinoculation except 100MOH and 50MOH that showed no effect against IBDV, thereby suggesting that the extracts have a preventive effect on CEF against viruses. During postinoculation, the extracts exhibited inhibitory effects against NDV and avian reovirus, while no effect against IBDV recorded and only the 100MOH showed an inhibitory effect against ILTV. The initial results of this study suggest that *Illicium verum* may be a candidate for a natural alternative source for antiviral agents.

## 1. Introduction

In spite of tremendous progress in the means of care, using medicine and vaccination to control infectious diseases, there is still a significant threat to the poultry industry. Viruses are one of the biggest causative agents of poultry diseases and are considered the principal risk to the poultry industry resulting in enormous economic losses to the poultry industry worldwide. Vaccination remains an important strategy to combat infection of poultry diseases. However, the infectious disease occurs despite vaccination against these diseases while antiviral agents are not used in poultry diseases due to toxicity and high costs of antiviral compounds [1, 2]. Therefore, it is necessary to find other ways to control viral diseases.

Currently, attention is increased in searching for new effective and safe compounds to control viral diseases in human beings and animals. A number of investigations were carried out to test different plant extracts against influenza virus (H1N1) [3], Newcastle disease virus [4], rotavirus [5], and herpes simplex virus type 1 [6]. Natural resources such as herbs and their extract were investigated for the biological action that gets them an encouraging prospect for research to control such disease. Star anise (*Illicium verum*) represents one of the medicinal herbs, especially after the popularity it achieved as an antiflu drug. Shikimic acid extracted from the fruits of star anise being used for the production of Tamiflu [7]. *Illicium verum* plant is an evergreen plant, usually recognized as Chinese star anise, which has star-shaped fruits and originates in China and Vietnam, traditionally practiced as a spice and herb. Also, it has medicinal properties that have significant health benefits [8]; star anise extracts showed antiviral activity against human immunodeficiency virus (HIV) [9], herpes simplex virus type 1 (HSV-1) [10], herpes simplex virus type 2 (HSV-2) [11], and bovine herpes virus type 1 (BHV-1) [12]. However, the data available about its activity against avian viruses are limited. This study was planned to evaluate the antiviral activity of the three extracts from *Illicium verum* against four avian viruses.

## 2. Material and Methods

### 2.1. The Plant

*Illicium verum* dry fruits were purchased from a local herb store in Riyadh, Saudi Arabia. The dry fruits of star anise were ground into a fine powder.

### 2.2. Preparation of Star Anise Extracts

The extraction of Chinese star anise was carried out using three solvents: absolute methanol, 50% methanol, and sterile distilled water. For each extract, 100 g of anise powder was added separately to 500 ml of the corresponding solvent and mixed well in tightly sealed flasks and then placed in a water bath at 37°C for 24 hours with intermittent shaking. The supernatant was collected and filtered (Whatman filter paper no. 1). The residue was kept in the flasks and the extraction process was repeated 3–5 times until a clear solution is obtained. After that, the extract solutions were collected and filtered again (Whatman no. 1). The extract was allowed to dry in the oven at 45°C. The dry extract was weighed and either diluted to a final concentration of 500 mg/ml or kept in fastened capped bottles in the refrigerator for subsequent usage. The diluted extracts were centrifuged and filtered through a 0.22 *μ*m Millipore membrane filter (Whatman, Kent, UK).

### 2.3. Cells and Viruses

Chick embryo fibroblast cells (CEF) were used in this study, and the cells were prepared from 9- to 11-day-old embryo chicks. Virus vaccine strains, avian reovirus (S1133), NDV (Clone 30), IBDV (D78), and ILTV (LT-IVAX) (Intervet Inc., Omaha NE68103 USA), were used in this study to assess the antiviral action of Chinese star anise extracts.

### 2.4. Preparation of Chick Embryo Fibroblast Culture

CEF cells were organized from 9- to 10-day-old chick embryo following the standard protocol described previously [13].

### 2.5. Propagation of Viruses

CEF monolayers grew in M199 medium supplemented with 10% newborn fetal serum and incubated at 37°C with 5% CO_2_. When the confluent CEF monolayers were obtained, they were subcultured using trypsin/EDTA solution. Once the new CEF grew to 85% confluence in T75 flasks (Corning), the growth medium was removed and 1 ml of each virus suspension stock was inoculated into the tissue culture flasks; the inoculated flasks were incubated for 1 hour at 37°C to allow viral adsorption. The virus suspensions were removed and M199 supplemented with 5% newborn calf serum was added and then the inoculated cells were incubated at 37°C and 5% CO_2_ for seven days and tested daily for cytopathic effect (CPE) using an inverted optical microscope (Nikon, Japan). The viruses were passaged five times to ensure adoption to CEF. Virus titration was done according to the method of Reed and Muench [14].

### 2.6. Cytotoxicity Assay

Cytotoxicity of *Illicium verum* extracts was evaluated in vitro using a cell viability assay known as MTT (3-(4, 5-dimethylthiazol-2-yl)-2,5-diphenyl tetrazolium bromide) method [15]. Confluent monolayers of CEF cells in 96-well tissue culture plates were incubated with different concentrations of *Illicium verum* extracts (250–0.48 mg/ml) diluted with M199 medium and incubated at 37°C with 5% CO_2_; cells without the extracts were applied as the control. After 72 hours of incubation, 20 *μ*l of MTT solution (5 mg/ml PBS) (Sigma) was added to each well, the plates were further incubated at 37°C for 4 hours, and then, the medium was removed and 100 *μ*l of dimethyl sulfoxide (DMSO) was added to each well. The plates were shaken in a microplate shaker for 5 minutes, and the absorbance was read at a wavelength of 570 nm using a microplate reader. The experiments were performed in duplicate and repeated three times.

### 2.7. Infection and Viral Growth Assay

The inhibitory antiviral activity of the extracts was evaluated by the ability of the extracts to inhibit CPE of viruses in CEF cells. According to the outcome of the cytotoxicity assay, the extracts were diluted into different concentrations starting from the maximum noncytotoxic concentration with M199 (3.9–0.24 mg/ml). The antiviral activity of *Illicium verum* extracts against avian reovirus, NDV, IBDV, and ILTV was evaluated in three approaches of addition following the method of Fan et al. [4] and Xu et al. [16]. In the first method, simultaneous inoculation, 100 TCID_50_ of virus suspensions was incubated together with *Illicium verum* extracts outside of cell culture for 1 hour at 4°C, and then the treated virus was added to the cell culture and incubated for 1 hour at 37°C. After that, virus suspensions were removed and the cells were washed with PBS and new M199 was added to the plates, and all plates were incubated at 37°C with 5% CO_2_; positive control wells contain cells and virus without the extracts, negative control wells included only cells in the medium, and blank wells contain only the medium. The cells were examined daily using an inverted optical microscope. In the preinoculation method, the *Illicium verum* extracts were incubated with the CEF cells for 2 hours at 37°C and 5% CO_2_, and then the extracts were removed and the treated cells were washed with PBS, and then 100 TCID_50_ of virus suspensions was incubated with the treated cells for 1 h. After that, virus suspensions were removed and maintenance medium was added to the plates, and the plates were incubated at 37°C in a humidified atmosphere and 5% CO_2_. The plates were then examined daily for CPE. In the postinoculation method, 100 TCID_50_ of the virus was incubated with monolayers of the CEF in 96-well plates and incubated for 1 hour at 37°C to allow attachment. After that, the medium was removed and the cells were washed with PBS, and then different concentrations of the extracts (3.9–0.24 mg/ml) were added to each well of the plates. The MTT assay was used to measure the livability of CEF, and the test is done when active control cells showed 80% CPE. The experiments were repeated three times. The virus inhibitory rate was calculated based on the following formula: virus inhibitory rate = ((extract + virus − virus control)/(cell control − virus control)) *∗* 100. The absorbance values and virus inhibitory rate were considered as indicators of antiviral activity.

### 2.8. Statistical Analysis

Data were analyzed by ANOVA using the GLM model of SAS® [17]. The Duncan multiple range test was used to compare differences among treatment means (*P* ≤ 0.05) when significant. Percentage data were subjected to arcsine transformation before analysis. All experiments were repeated three times.

## 3. Results and Discussion

In the cytotoxicity assay using MTT, the absorbance value of cells is used as an indicator of the number of living cells and related to cell growth [18], so that high absorbance value means more living cells. When the absorbance values of the treated cells were not significantly lower than cell control, it indicated that the extract had no cytotoxicity on cells, and the corresponding concentration could consider as the maximal safety level. The results of the cytotoxicity test of *Illicium verum* extracts are listed in Table 1. The results showed that the 100MOH and 50MOH extracts at 3.9 mg/ml and WA extract at 1.9 mg/ml were not significantly lower than those of the cell control group. Therefore, these concentrations were considered as the maximal safety levels. The 100MOH and 50MOH extracts at high concentrations (62.5 to 7.8 mg/ml) were toxic to CEF as indicated by the lower value of absorbance compared to cell control. The aqueous extract at a concentration of 62.5 mg/ml showed no significant difference in comparison with cell control. The high absorbance value may be due to the darker color of the aqueous extract at this high concentration (62.5 mg/ml), but at the following levels (31.25–3.9 mg/ml), the absorbance values were significantly lower than the control group. On the other side, the aqueous extract at the concentrations 1.9 and 0.97 mg/ml exhibited significantly larger values (*P* ≤ 0.05) than the control group that may indicate that this extract could promote the cell growth within these dose ranges [4]. For facilitating the comparison between the extracts at the same levels, the maximal safety concentration of the extracts was considered at 3.9 mg/ml.

The antiviral activity of *Illicium verum* extracts against IBDV is presented in Table 2. In preinoculation, the 100MOH and 50MOH extracts showed no significant difference from the corresponding virus control group at all concentrations. The aqueous extract at 0.48 and 0.24 mg/ml exhibited significantly (*P* ≤ 0.05) more considerable value than the virus control group, which indicated that the aqueous extract could prevent IBDV infection at a particular concentration. During postinoculation, the three extracts showed no significant difference from the corresponding virus control group at all levels, which indicated that the extracts have no treatment effect against IBDV after infection. In simultaneous inoculation, the values of 100MOH extract at 0.48 and 0.24 mg/ml, 50MOH at 0.48 mg/ml, and WA extracts at 1.9 to 0.48 mg/ml were significantly (*P* ≤ 0.05) larger than those of the corresponding virus control group, which indicated that the three extracts could have virucidal effect against IBDV at specific dose.

**Figure 1:**
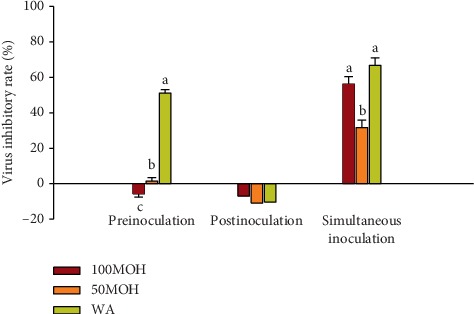
Virus inhibitory rate of *Illicium verum* extracts against infectious bursal disease virus using three adding methods. 100MOH = absolute methanol extract; 50MOH = 50% methanol extract; WA = water extract.

The virus inhibitory rates of the extracts against IBDV are illustrated in Figure 1. The extracts showed inhibitory activity against IBDV during simultaneous inoculation, and 100MOH and WA presented the highest (*P* ≤ 0.05) inhibitory rates of 56% and 67%, respectively. The 50MOH extract showed the lowest inhibitory rate (32%), while in postinoculation, no inhibitory effect was detected in the three extracts, and only the WA extract showed an inhibitory effect against IBDV in preinoculation mode (51%). Based on these results, the WA extract has a significant antiviral effect in two adding methods (preinoculation and simultaneous), while the other extracts have a significant antiviral effect only in simultaneous process, and hence, the antiviral activity of WA extract against IBDV was better than that of other extracts.

The antiviral activity of *Illicium verum* extracts against ILTV is illustrated in Table 3. During preinoculation, the 100MOH extract at 0.48 and 0.24 mg/ml and WA extract at 0.97 to 0.24 mg/ml concentrations showed significantly (*P* ≤ 0.05) higher values compared to the virus control group, while no significant difference was found between the 50MOH extract and virus control group (*P* ≤ 0.05), which indicated that the 100MOH and aqueous extracts could prevent ILTV infection at a particular concentration. In postinoculation, the 50MOH and WA extracts showed no significant difference with the corresponding virus control group at all levels, while the 100MOH exhibited significantly (*P* ≤ 0.05) higher value compared to the virus control group, which revealed that the 100MOH extract could treat ILTV infection. In simultaneous inoculation, the 100MOH extract at 3.9 and 1.9 mg/ml, 50MOH at 3.9 to 0.48 mg/ml, and WA extract at all concentrations were significantly (*P* ≤ 0.05) higher than those of the corresponding virus control groups, which indicated that the three extracts could have virucidal activity against ILTV at a particular dose.

**Figure 2:**
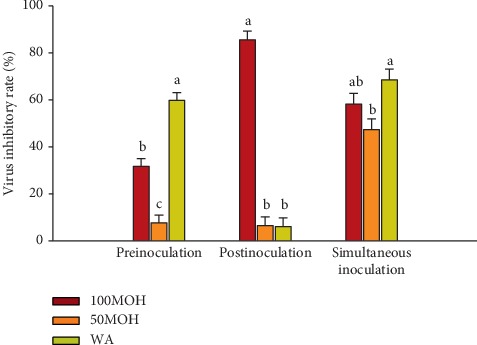
Virus inhibitory rate of *Illicium verum* extracts against infectious laryngotracheitis virus using three adding methods. 100MOH = absolute methanol extract; 50MOH = 50% methanol extract; WA = water extract.

The virus reduction rates of the extracts against ILTV are shown in Figure 2. During preinoculation, WA extract exhibited the highest (*P* ≤ 0.05) virus inhibitory rate (60%), followed by the 100MOH with 32% inhibitory rate, while the 50MOH showed the lowest inhibitory rate (8%) against ILTV. During postinoculation modes, only 100MOH exhibited inhibitory activity against ILTV with the highest reduction rate (85%). In simultaneous inoculation, WA extract presented the most elevated (*P* ≤ 0.05) inhibitory rate (69%) while no significant differences were found between 100MOH (52%) and 50MOH extracts (47%). These results showed that the 100MOH extract had a significant (*P* ≤ 0.05) antiviral activity against ILTV in the three adding methods, WA extract has antiviral activity in two adding models (preinoculation and simultaneous) while 50MOH extract had such activity only in simultaneous process. Therefore, the antiviral activity of 100MOH and WA extracts against ILTV was better than that of 50MOH extract. Furthermore, 100MOH had to treat effect after ILTV infection, while the other extracts did not show such effect.

The antiviral activity of *Illicium verum* extracts against NDV is summarized in Table 4. All extracts showed a preventive effect against NDV infection at specific concentrations at preinoculation. The 100MOH extract at 0.24 mg/ml, 50MOH at 0.48 mg/ml, and WA extract at all concentrations showed significantly higher values compared to the virus control group (*P* ≤ 0.05). Also, there was no significant difference between the WA extract at 0.48 and 0.24 mg/ml compared to the cell control group, which indicated a high protective effect of the WA extract at these concentrations against the inactivity of NDV. Similar results were found in postinoculation modes, and the three extracts exhibited a treatment effect against NDV. After infection, the 100MOH, 50MOH (0.48 and 0.24 mg/ml), and WA extracts (0.97 and 0.48 mg/ml) showed significantly (*P* ≤ 0.05) higher difference compared with the virus control group. In simultaneous inoculation, the values of 100MOH extract at 0.97 mg/ml, 50MOH at 0.48 and 0.24 mg/ml, and WA extracts at 0.97 to 0.24 mg/ml concentrations were higher (*P* ≤ 0.05) than those of the corresponding virus control groups which indicated that the three extracts could have virucidal activity against NDV at certain doses.

**Figure 3:**
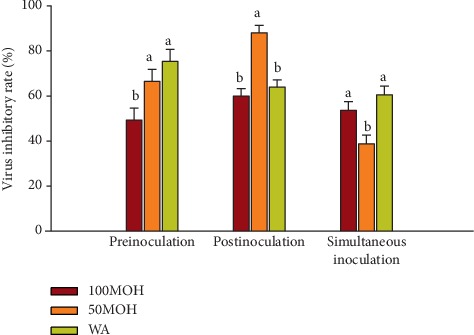
Virus inhibitory rate of *Illicium verum* extracts against Newcastle disease virus using three adding methods. 100MOH = absolute methanol extract; 50MOH = 50% methanol extract; WA = water extract.

The virus inhibitory rates of the three extracts against NDV are presented in Figure 3. In preinoculation, WA and 50MOH extracts demonstrated the highest (*P* ≤ 0.05) virus inhibitory rate (75% and 66%, respectively), and the 100MOH showed the lowest inhibitory rate (49%) against NDV. In the postinoculation method, 50MOH exhibited the highest (*P* ≤ 0.05) reduction rate (88%), while 100MOH and WA extracts showed a similar reduction effect, 60%, and 63%, respectively. After simultaneous inoculation, the WA (60%) and 100MOH (53%) extracts exhibited the highest reduction rate, while 50MOH extract showed the lowest inhibitory rate (38%). These results showed that the extracts had significant antiviral activity against NDV in three inoculation methods.

The results of the antiviral activity of *Illicium verum* extracts against avian reovirus are illustrated in Table 5. The three extracts revealed preventive action against reovirus infection at specific concentrations during preinoculation modes. The 100MOH extract and 50MOH at 1.9–0.48 mg/ml and WA extract from 3.9 to 0.48 mg/ml showed significantly (*P* ≤ 0.05) higher values compared to the virus control group (*P* ≤ 0.05). The 100MOH extract at 1.9 to 0.48 mg/ml showed no significant difference compared to the control cell group, indicating a high protective effect on CEF against avian reovirus infection. During postinoculation modes, the three extracts exhibited a treatment effect against avian reovirus infection at a particular dose, and the values of 100MOH at 0.97 mg/ml, 50MOH at 0.24 mg/ml, and WA extracts at 0.97–0.24 mg/ml were significantly (*P* ≤ 0.05) higher than those of the virus control group. In simultaneous inoculation, the values of 50MOH at 0.48 mg/ml and WA extracts at 0.24 mg/ml concentrations were significantly higher (*P* ≤ 0.05) than those of the virus control groups, which indicated that these extracts could have a virucidal effect on avian reovirus at an appropriate dose.

**Figure 4:**
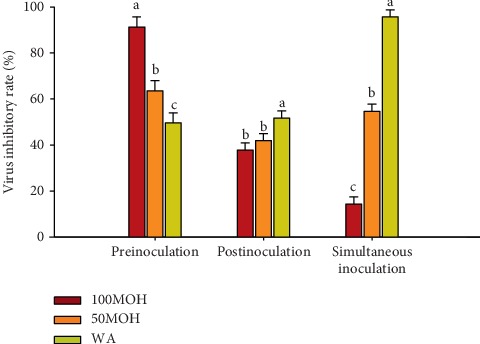
Virus inhibitory rate of *Illicium verum* extracts against avian reovirus using three adding methods. 100MOH = absolute methanol extract; 50MOH = 50% methanol extract; WA = water extract.

The virus inhibitory rates of the three extracts against avian reovirus are presented in Figure 4. In preinoculation, 100MOH extract revealed the highest (*P* ≤ 0.05) virus inhibitory rate (*P* ≤ 0.05) against avian reovirus (91%), followed by 50MOH with a 63% reduction rate, while the WA extract showed the lowest inhibitory rate (49%). During the postinoculation method, the WA extract exhibited the highest reduction rate (51%), while 100MOH and 50MOH extracts showed a similar reduction effect with inhibitory rates of 38% and 42%, respectively. After simultaneous inoculation, the WA extract presented the highest (*P* ≤ 0.05) reduction effect against avian reovirus (95%), 50MOH extracts showed 55% reduction rate, and 100MOH extract showed the lowest inhibitory rate (14%). These results indicated that both WA and 50MOH extracts had significant antiviral activity against avian reovirus in the three adding methods while the 100MOH extract had high antiviral activity against avian reovirus during the preinoculation mode.

In general, the three adding methods of the extracts and tested viruses refer to the stages of the virus infection cycle. Results showed that the extracts exhibited antiviral activity against all tested viruses during simultaneous inoculation with respect to reduction rate among each extract on each virus, indicating that the extracts have an inhibitory effect during before virus adsorption or attachment to CEF cells. During preinoculation, similar findings were mentioned except 100MOH and 50MOH extract against IBD, which may indicate that the extracts are interfering with virus attachment and penetration of host cells, suggesting that the extracts have a preventive effect on CEF against viruses. However, in postinoculation, the results showed the different impact of the extracts in each virus; the extracts exhibited antiviral effect against NDV and reovirus while no effect against IBDV and only the 100MOH extract showed activity against ILTV; these results indicate that anise extracts have virucidal properties and may prevent the virus replication after infection. The differences among viruses in response to *Illicium verum* extracts in this study may be attributed to the differences in the levels of active components in the extracts and variations among the tested viruses. For example, IBDV is a nonenveloped and highly resistant virus [19]. It was proposed that the antiviral action of plant extracts may be ascribable to an interaction with the viral envelopes [20, 21]. However, the mechanism of action is still not clear, whether the inhibitory effect of anise extracts is due to interference with cellular membrane proteins or virus receptors involved in host cell adsorption and penetration, consequently preventing virus infection of the cells [11, 22]. The results of our study refer that the inhibitory effect of *Illicium verum* may be due to not only the essential oils but also other elements that contributed to reducing the infectious ability of viruses. For instance, lignin-carbohydrate complexes, isolated by hot water extraction from seeds of *Pimpinella anisum*, exhibited antiviral activities against HSV-I and HSV-2, human cytomegalovirus (HCMV), and measles virus [23]. Also, these compounds interfere with virus adsorption to the host cell surface and inactivate viruses. Schnitzler et al. [24] indicated that palm oil, abundant in phenolic compounds, could affect the infectivity of herpes viruses in vitro when added before viral adsorption by binding to the viral proteins involved in the host adsorption and penetration. The results of this work are in agreement with other previous studies. The compounds (-)-illicinone-A, 3,4-seco-(24Z)-cycloart-4(28), 24-diene-3,26-dioic acid, and 26-methyl ester extracted from *Illicium verum* roots displayed moderate anti-HIV activity [9]. *Illicium verum* essential oil inhibited viral infectivity by 99%, while phenylpropanoids reduced the HSV infectivity by 60–80% and sesquiterpenes inhibited the infectivity by 40–98% [10]. In another study, anise oil showed dose-dependent antiviral activity against HSV-2 [25]. There was no inhibitory effect when the essential oils were added to the cells before infection or after the adsorption period. The authors concluded that essential oils may interact with the virus envelope and prevent the adsorption of the virus.

In conclusion, the three extracts showed antiviral activity against all tested viruses during simultaneous inoculation and preinoculation except 100MOH and 50MOH that showed no effect against IBD, thereby indicating that the extracts have a prophylactic effect on CEF against viruses. Nevertheless, in postinoculation, the extracts exhibited inhibitory effects against NDV and avian reovirus, while no outcome found against IBDV and only the 100MOH showed an inhibitory effect against ILTV. The initial results of this study suggest that *Illicium verum* may be a candidate for a natural alternative source for antiviral agents. However, more in vivo trials are required to confirm these findings.

## Figures and Tables

**Table 1:** Absorbance means of the cytotoxicity of *Illicium verum* extracts.

Concentration (mg/ml)	Star anise extracts^1^		
	100MOH	50MOH	WA
62.5	0.152^c^	0.198^d^	0.379^c^
31.25	0.126^d^	0.141^e^	0.293^e^
15.62	0.108^d^	0.124^e^	0.247^f^
7.8	0.116^d^	0.258^c^	0.127^g^
3.9	0.390^a^	0.361^a^	0.324^d^
1.9	0.382^a^	0.324^b^	0.458^a^
0.97	0.389^a^	0.327^b^	0.410^b^
0.48	0.353^b^	0.307^b^	0.373^c^
Cell control	0.402^a^	0.380^a^	0.395^bc^
SEM	0.007	0.009	0.009
*P* value	<0.0001	<0.0001	<0.0001

Means within a column with different subscripts are significantly different (*P* ≤ 0.05). ^1^100MOH = absolute methanol extract; 50MOH = 50% methanol extract; WA = water extract.

**Table 2:** Absorbance means of the antiviral activity of *Illicium verum* extracts against infectious bursal disease virus in the three adding methods.

Concentrations (mg/ml)	Preinoculation			Postinoculation			Simultaneous inoculation		
	100MOH	50MOH	WA	100MOH	50MOH	WA	100MOH	50MOH	WA^1^
3.9	0.181^b^	0.185^b^	0.185^c^	0.183^b^	0.164^b^	0.172^b^	0.205^d^	0.198^d^	0.277^d^
1.9	0.183^b^	0.195^b^	0.193^c^	0.192^b^	0.169^b^	0.191^b^	0.187^d^	0.206^d^	0.338^c^
0.97	0.184^b^	0.183^b^	0.220^c^	0.197^b^	0.178^b^	0.181^b^	0.226^cd^	0.216^d^	0.343^bc^
0.48	0.195^b^	0.190^b^	0.303^b^	0.168^b^	0.167^b^	0.168^b^	0.356^b^	0.325^b^	0.391^b^
0.24	0.216^b^	0.229^b^	0.321^b^	0.181^b^	0.191^b^	0.189^b^	0.374^b^	0.292^bc^	0.288^d^
Virus control	0.226^b^	0.226^b^	0.226^c^	0.211^b^	0.211^b^	0.211^b^	0.265^c^	0.265^c^	0.265^d^
Cell control	0.412^a^	0.412^a^	0.412^a^	0.409^a^	0.409^a^	0.409^a^	0.458^a^	0.458^a^	0.458^a^
*P* value	<0.0001	<0.0001	<0.0001	<0.0001	<0.0001	<0.0001	<0.0001	<0.0001	<0.0001
SEM	0.01	0.01	0.02	0.01	0.01	0.01	0.01	0.01	0.01

Means within a column with different subscripts are significantly different (*P* ≤ 0.05). ^1^100MOH = absolute methanol extract; 50MOH = 50% methanol extract; WA = water extract.

**Table 3:** Absorbance means of the antiviral activity of *Illicium verum* extracts against infectious laryngotracheitis virus in the three adding methods.

Concentrations (mg/ml)	Preinoculation			Postinoculation			Simultaneous inoculation		
	100MOH	50MOH	WA	100MOH	50MOH	WA	100MOH	50MOH	WA^1^
3.9	0.187^c^	0.181^b^	0.208^c^	0.256c	0.217^b^	0.195^b^	0.349^b^	0.308^b^	0.325^b^
1.9	0.186^c^	0.185^b^	0.193^c^	0.353b	0.199^b^	0.217^b^	0.271^c^	0.317^b^	0.357^b^
0.97	0.194^c^	0.187^b^	0.270^b^	0.335b	0.203^b^	0.206^b^	0.206^d^	0.303^b^	0.358^b^
0.48	0.249^b^	0.196^b^	0.301^b^	0.374ab	0.217^b^	0.196^b^	0.196^d^	0.318^b^	0.369^b^
0.24	0.260^b^	0.204^b^	0.316^b^	0.265c	0.201^b^	0.198^b^	0.173^d^	0.246^c^	0.325^b^
Virus control	0.186^c^	0.186^b^	0.186^c^	0.204d	0.204^b^	0.204^b^	0.198^d^	0.198^c^	0.198^c^
Cell control	0.412^a^	0.412^a^	0.412^a^	0.409a	0.409^a^	0.409^a^	0.458^a^	0.458^a^	0.458^a^
*P* value	<0.0001	<0.0001	<0.0001	<0.0001	<0.0001	<0.0001	<0.0001	<0.0001	<0.0001
SEM	0.02	0.01	0.02	0.02	0.01	0.01	0.02	0.02	0.02

Means within a column with different subscripts are significantly different (*P* ≤ 0.05). ^1^100MOH = absolute methanol extract; 50MOH = 50% methanol extract; WA = water extract.

**Table 4:** Absorbance means of the antiviral activity of *Illicium verum* extracts against Newcastle disease virus in the three adding methods.

Concentrations (mg/ml)	Preinoculation			Postinoculation			Simultaneous inoculation		
	100MOH	50MOH	WA	100MOH	50MOH	WA	100MOH	50MOH	WA^1^
3.9	0.311^bc^	0.297^c^	0.361^b^	0.174^d^	0.209^b^	0.193^d^	0.188^d^	0.190^d^	0.233^c^
1.9	0.289^bc^	0.319^bc^	0.342^b^	0.216^c^	0.197^b^	0.278^bc^	0.269^c^	0.227^cd^	0.230^c^
0.97	0.320^bc^	0.301c	0.361^b^	0.249^c^	0.206^b^	0.314^b^	0.349^b^	0.281^bc^	0.330^b^
0.48	0.322^bc^	0.384^b^	0.387^ab^	0.318^b^	0.348^a^	0.323^ab^	0.298^bc^	0.312^b^	0.359^b^
0.24	0.356^b^	0.320^bc^	0.402^ab^	0.317^b^	0.352^a^	0.249^c^	0.196^d^	0.316^b^	0.363^b^
Virus control	0.255^c^	0.255^c^	0.255^c^	0.237^c^	0.237^b^	0.237^cd^	0.242^cd^	0.242^c^	0.242^c^
Cell control	0.454^a^	0.454^a^	0.454^a^	0.367^a^	0.367^a^	0.367^a^	0.445^a^	0.445^a^	0.445^a^
*P* value	0.0004	0.0004	0.0007	<0.0001	<0.0001	<0.0001	<0.0001	<0.0001	0.0003
SEM	0.02	0.02	0.02	0.01	0.01	0.01	0.02	0.02	0.02

Means within a column with different subscripts are significantly different (*P* ≤ 0.05). ^1^100MOH = absolute methanol extract; 50MOH = 50% methanol extract; WA = water extract.

**Table 5:** Absorbance means of the antiviral activity of *Illicium verum* extracts against the avian reovirus in the three adding methods.

Concentrations (mg/ml)	Preinoculation			Postinoculation			Simultaneous inoculation		
	100MOH	50MOH	WA	100MOH	50MOH	WA	100MOH	50MOH	WA^1^
3.9	0.294^c^	0.326^bc^	0.356^b^	0.182^d^	0.205^d^	0.228^c^	0.243^c^	0.199^d^	0.184^d^
1.9	0.434^a^	0.388^b^	0.354^b^	0.188^d^	0.200^d^	0.225^c^	0.301^bc^	0.288^c^	0.336^c^
0.97	0.412^a^	0.349^b^	0.358^b^	0.285^b^	0.195^d^	0.277^b^	0.322^b^	0.356^b^	0.356^bc^
0.48	0.387^ab^	0.378^b^	0.367^b^	0.233^c^	0.241^c^	0.303^b^	0.262^bc^	0.382^b^	0.411^ab^
0.24	0.330^bc^	0.332^bc^	0.331^bc^	0.242^c^	0.291^b^	0.304^b^	0.242^bc^	0.371^b^	0.440^a^
Virus control	0.282^c^	0.282^c^	0.282^c^	0.235^c^	0.235^c^	0.235^c^	0.301^bc^	0.301^c^	0.301^c^
Cell control	0.454^a^	0.454^a^	0.454^a^	0.367^a^	0.367^a^	0.367^a^	0.445^a^	0.445^a^	0.445^a^
*P* value	0.0007	0.001	0.006	<0.0001	<0.0001	<0.0001	0.0009	<0.0001	<0.0001
SEM	0.02	0.01	0.02	0.01	0.009	0.01	0.02	0.02	0.02

Means within a column with different subscripts are significantly different (*P* ≤ 0.05). ^1^100MOH = absolute methanol extract; 50MOH = 50% methanol extract; WA = water extract.

## Data Availability

The data used to support the findings of this study are included within the article.
